# Phosphodiesterase Type 5 Inhibitors in Male Reproduction: Molecular Mechanisms and Clinical Implications for Fertility Management

**DOI:** 10.3390/cells14020120

**Published:** 2025-01-15

**Authors:** Aris Kaltsas, Fotios Dimitriadis, Athanasios Zachariou, Nikolaos Sofikitis, Michael Chrisofos

**Affiliations:** 1 Third Department of Urology, Attikon University Hospital, School of Medicine, National and Kapodistrian University of Athens, 12462 Athens, Greece; ares-kaltsas@hotmail.com; 2 Department of Urology, Faculty of Medicine, School of Health Sciences, Aristotle University of Thessaloniki, 54124 Thessaloniki, Greece; helabio@yahoo.gr; 3 Laboratory of Spermatology, Department of Urology, Faculty of Medicine, School of Health Sciences, University of Ioannina, 45110 Ioannina, Greece; azachariou@uoi.gr (A.Z.); nsofikit@uoi.gr (N.S.)

**Keywords:** phosphodiesterase (PDE), PDE5 inhibitors, male reproduction, spermatogenesis, sperm motility, testicular physiology, andrology, reproductive endocrinology

## Abstract

Phosphodiesterases, particularly the type 5 isoform (PDE5), have gained recognition as pivotal regulators of male reproductive physiology, exerting significant influence on testicular function, sperm maturation, and overall fertility potential. Over the past several decades, investigations have expanded beyond the original therapeutic intent of PDE5 inhibitors for erectile dysfunction, exploring their broader reproductive implications. This narrative review integrates current evidence from in vitro studies, animal models, and clinical research to clarify the roles of PDEs in effecting the male reproductive tract, with an emphasis on the mechanistic pathways underlying cyclic nucleotide signaling, the cellular specificity of PDE isoform expression, and the effects of PDE5 inhibitors on Leydig and Sertoli cell functions. Although certain findings suggest potential improvements in sperm motility, semen parameters, and a more favorable biochemical milieu for spermatogenesis, inconsistencies in study design, limited sample sizes, and inadequate long-term data temper definitive conclusions. Addressing these gaps through standardized protocols, larger and more diverse patient cohorts, and explorations of mechanistic biomarkers could pave the way for incorporating PDE5 inhibitors into evidence-based fertility treatment strategies. In the future, such targeted approaches may inform individualized regimens, optimize male reproductive outcomes, and refine the clinical application of PDE5 inhibitors as part of comprehensive male fertility management.

## 1. Introduction

Male infertility has emerged as a significant public health concern, affecting an estimated 7% of men worldwide and driving increased reliance on assisted reproductive technologies [1]. While considerable progress has been made in diagnostic techniques and therapeutic interventions, many treatment strategies still yield variable results, reflecting an incomplete grasp of the complex molecular and physiological processes governing sperm production and function [2]. Advancing our understanding of male reproductive biology is therefore essential for developing more effective, targeted approaches to fertility management.

Within this evolving landscape, phosphodiesterase type 5 (PDE5) inhibitors, originally developed for and extensively validated in the treatment of erectile dysfunction, have begun to receive attention for their potential roles beyond penile hemodynamics [3]. Phosphodiesterases (PDEs), which regulate intracellular levels of cyclic nucleotides, are increasingly recognized as key modulators of testicular and accessory gland function. Multiple PDE isoforms are expressed throughout the male reproductive tract, influencing spermatogenesis, sperm maturation, and the orchestration of events critical to fertilization [4]. Notably, some studies suggest that PDE5 inhibitors could enhance semen parameters such as sperm motility—the sperm’s capacity for directional movement—and improve capacitation, a post-ejaculatory transformation required for successful oocyte penetration. These enhancements could, in theory, bolster overall fertilization potential, or the ability of sperm to achieve fertilization [5]. However, reports remain inconsistent, with some studies noting modest benefits while others question their net impact, including potential alterations in hormone homeostasis [6].

Given these emerging but inconclusive findings, a thorough exploration of PDE-mediated pathways in the male reproductive system and the precise influence of PDE5 inhibitors on these pathways is both timely and needed. This review synthesizes current evidence from molecular research, animal models, and clinical studies to clarify the mechanistic roles of PDEs, highlight how PDE5 inhibitors might influence male fertility, and identify critical knowledge gaps. By doing so, it aims to guide future research efforts, pave the way for evidence-based clinical strategies, and ultimately improve fertility outcomes for affected individuals and couples.

## 2. Mechanisms of Action of Phosphodiesterases in Cyclic Nucleotide Regulation

PDEs regulate intracellular signaling by hydrolyzing the second messengers cyclic adenosine monophosphate (cAMP) and cyclic guanosine monophosphate (cGMP), thereby controlling their concentration and activity in a cell-specific, temporally dynamic, and spatially precise manner [7]. The PDE superfamily comprises eleven families (PDE1–PDE11), encoded by 21 distinct genes. Through the use of alternative promoters and mRNA splicing, these genes produce over 50 isoforms with diverse tissue distributions and regulatory mechanisms [8,9]. PDE isoforms are categorized based on their amino acid sequences, substrate specificity, cofactor dependencies, catalytic activity, and kinetic properties. All PDEs share a conserved catalytic domain (C domain) but exhibit variability in their N-terminal regulatory domains, which govern isoform-specific functions and interactions [9]. Certain PDE families selectively hydrolyze cAMP (e.g., PDE4, PDE7, PDE8) or cGMP (e.g., PDE5, PDE6, PDE9), whereas others, such as PDE1, PDE2, PDE3, PDE10, and PDE11, act on both cyclic nucleotides, thus orchestrating a wide spectrum of physiological processes [10].

### 2.1. Regulatory Mechanisms of PDEs

The activity of PDEs is modulated by several mechanisms, enabling precise control of cyclic nucleotide levels in response to dynamic cellular cues. These regulatory mechanisms include the following: (i) The binding of Ca^2+^/calmodulin, as observed in PDE1. (ii) Phosphorylation and dephosphorylation processes that regulate the activity of enzymes such as PDE1, PDE3, PDE4, PDE5, and PDE8 by modifying their regulatory domains, thereby influencing substrate specificity and catalytic activity [11]. Not all such regulatory phosphorylations occur within N-terminal domains; for example, PDE4 is also regulated by ERK phosphorylation at its C-terminal end [12]. (iii) Allosteric interactions with GAF domains, which are characteristics of PDE2, PDE5, PDE6, PDE10, and PDE11, allowing these isoforms to modulate cyclic nucleotide signaling in a tissue- and cell-specific manner [9]. However, in contrast to the other PDEs mentioned, the PDE10 GAF binds and is regulated by cAMP, not cGMP [13]. By integrating these mechanisms, PDEs fine-tune signal transduction pathways that influence muscle contraction, ion transport, gene transcription, and numerous other essential cell functions.

### 2.2. The NO-cGMP-PKG Pathway

Nitric oxide (NO) is a key upstream regulator of cGMP signaling. Synthesized from L-arginine by neuronal, inducible, or endothelial nitric oxide synthases (NOS), NO binds intracellularly to soluble guanylyl cyclase (sGC), which converts guanosine triphosphate (GTP) into cGMP [14,15,16]. The resulting cGMP activates protein kinase G (PKG), which modulates ion channels and pumps, reducing calcium influx and promoting intracellular calcium sequestration. This cascade leads to smooth muscle relaxation and has critical ramifications for vascular tone, endothelial permeability, and cellular differentiation [17,18].

In the context of male reproductive physiology, the NO-cGMP-PKG pathway likely contributes to the regulation of testicular blood flow, thereby influencing nutrient delivery, oxygenation, and hormonal signaling essential for spermatogenesis. Similar mechanisms may also modulate contractility within the epididymis and efferent ducts, facilitating sperm transport and maturation. By modulating both vascular smooth muscle, such as that in the testicular microvasculature, and non-vascular smooth muscle activity, including the peritubular myoid cells of the seminiferous tubules and the contractile cells within the epididymal ducts, NO and cGMP signaling are poised to affect key reproductive events. These range from the maintenance of an optimal testicular microenvironment to the proper function of accessory glands [19].

In vascular smooth muscle cells, NO-synthesized cGMP activates PKG to promote vasodilation, while PDE5 counterbalances this effect by degrading cGMP [20]. In male reproductive tissues, an analogous interplay of NO-cGMP-PKG and PDE5 activity may help maintain the delicate equilibrium needed for processes such as seminiferous tubule fluid secretion, testicular contractility, and sperm passage through the male reproductive tract [21].

**Figure 1:**
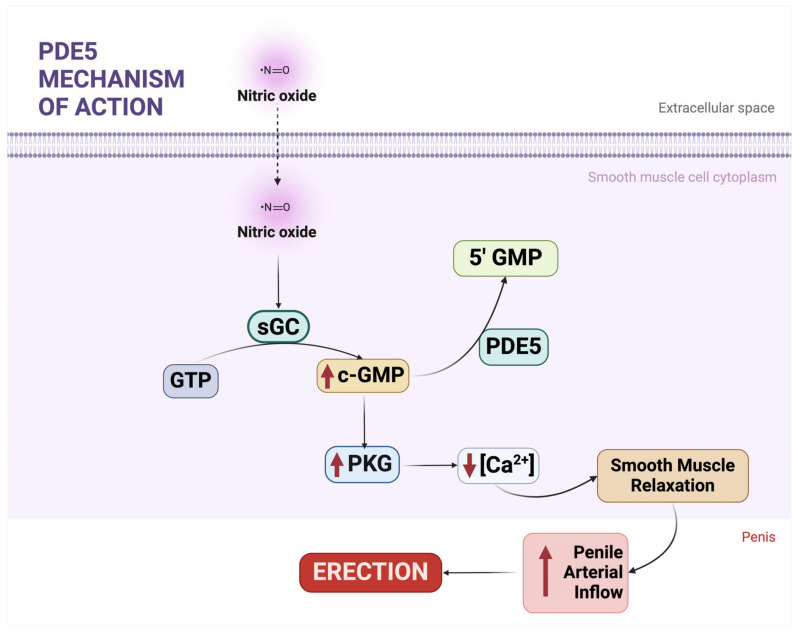
The mechanism of action of phosphodiesterase type 5 (PDE5). Guanosine triphosphate (GTP) is converted into cyclic guanosine monophosphate (cGMP) by soluble guanylyl cyclase (sGC) in response to nitric oxide (NO) signaling. cGMP activates protein kinase G (PKG), which regulates downstream processes such as calcium (Ca^2+^) handling and smooth muscle relaxation. PDE5 hydrolyzes cGMP into guanosine monophosphate (5′-GMP), thereby terminating the cGMP-mediated signaling pathway. This regulation maintains the balance of cyclic nucleotide signaling essential for vascular and reproductive physiology. Created in BioRender (https://BioRender.com/c37c727, accessed on 13 January 2025).

Figure 1 illustrates the mechanism of action of PDE5, highlighting its role in cGMP hydrolysis and the downstream effects of its inhibition, which elevate cGMP levels and enhance the signaling pathways essential for processes like smooth muscle relaxation and reproductive physiology.

### 2.3. PDE5 and Its Biological and Therapeutic Significance

The PDE5 family, encoded by the PDE5A gene, includes three splice variants: PDE5A1, PDE5A2, and PDE5A3. These isoforms share conserved phosphorylation sites, allosteric cGMP-binding domains, and catalytic regions but differ in their N-terminal regions and tissue distribution. PDE5A3 is predominantly expressed in vascular smooth muscle cells, while PDE5A1 and PDE5A2 are more broadly distributed [8]. 

PDE5 inhibitors, by preventing cGMP degradation, enhance erectile responses under sexual stimulation. Beyond facilitating penile erection, these agents exert anti-inflammatory, antioxidant, and antiproliferative effects, expanding their therapeutic relevance to conditions such as pulmonary and systemic hypertension [22]. Additionally, PDE5 inhibitors improve platelet function by increasing cGMP levels, thus augmenting the inhibitory influence of NO on platelet aggregation and activation [23].

### 2.4. Systemic Roles of PDE5

PDE5 plays significant roles outside the vascular system, affecting various organs and tissues through the modulation of cGMP signaling. In the kidneys, it modulates renal blood flow and renin production, while also influencing sodium excretion through cGMP-dependent natriuresis [24]. In the lungs, PDE5 is highly expressed and contributes to pulmonary vasodilation, making it a therapeutic target for conditions like pulmonary arterial hypertension [25].

In the heart, PDE5 influences cardiac function through cGMP-PKG signaling, although its expression in cardiomyocytes is debated. Its inhibition has shown potential cardioprotective effects in specific pathologies like heart failure [26]. Additionally, clinical evidence suggests that PDE5 inhibitors may benefit diastolic function by improving left ventricular relaxation and reducing cardiac filling pressures. In a recent prospective observational study, long-term tadalafil use was associated with a reduced E/e’ ratio and reduced pulmonary artery pressures, indicating enhanced diastolic indices in patients undergoing nerve-sparing robot-assisted radical prostatectomy [27]. While further large-scale research is required, these findings highlight the possible supportive role of PDE5 inhibition in heart failure management.

The eyes also demonstrate PDE5 activity, where it plays a role in ocular pressure regulation and retinal physiology [28]. Furthermore, PDE5 is present in neurons, where it participates in cGMP-mediated signaling pathways, suggesting its potential role in neurological conditions [29].

These systemic effects underscore the enzyme’s broad physiological importance. Within the male reproductive system, similar cGMP-dependent pathways regulated by PDE5 may be critical for ensuring proper testicular microcirculation, thereby indirectly supporting spermatogenesis and overall reproductive competence [30].

### 2.5. PDEs in Reproductive Tissues

PDEs play diverse roles in reproductive tissues, although their specific functions remain incompletely elucidated. Multiple isoforms, including PDE1C (which hydrolyzes both cAMP and cGMP [Km ≈ 1 μM]) and cAMP-specific PDEs such as PDE4A, PDE4C, PDE7B, and PDE8A, have been identified in testicular germ cells and spermatozoa. Additionally, PDE10, known for hydrolyzing cGMP, has been detected in the human testis [31,32]. In rodent studies, PDE5 has been localized in peritubular myoid cells, indicating its potential involvement in testicular physiology and the processes underpinning sperm maturation [33].

Beyond the testis, PDEs have roles in other reproductive tissues, including the epididymis, vas deferens, seminal vesicles, and prostate. In the epididymis, PDE5 contributes to the regulation of cGMP-mediated smooth muscle relaxation, which is critical for sperm transport and maturation [34]. In the vas deferens, PDE5 inhibitors have been shown to relax smooth muscle fibers, potentially modulating the ejaculation process [35].

In the seminal vesicles, PDE5 plays a role in regulating smooth muscle contractility, which impacts semen composition and ejection. In the prostate, PDE5 influences smooth muscle tone and secretory function, which are essential for proper sperm motility and male fertility [34].

Through these influences on smooth muscle tone, fluid homeostasis, and signal transduction, PDEs, and PDE5 in particular, are emerging as central players in the intricate regulation of male fertility [36].

## 3. Phosphodiesterases in the Male Reproductive System

PDEs are central regulators of cyclic nucleotide signaling in the male reproductive system, influencing processes such as sperm maturation and spermatogenesis. Studies in bovine and rat models have identified PDE-expressing contractile cells (smooth muscle cells and myofibroblasts) within the seminiferous tubules and along the epididymal and efferent ducts. These cells facilitate the transport of immotile testicular spermatozoa to the cauda epididymis, where sperm ultimately acquire motility and fertilization capacity [37,38,39,40]. Human data further indicate that alterations in the peritubular lamina propria, including an increase in contractile cells and extracellular matrix components, may underlie certain cases of idiopathic male infertility. This is supported by experimental models, where deletion of the androgen receptor gene specifically in peritubular muscle cells results in impaired spermatogenesis. It is important to clarify that while the androgen receptor is not an extracellular component, its expression in the peritubular cells influences contractile and extracellular matrix dynamics, potentially contributing to testicular dysfunction and reduced spermatogenesis [41,42].

With the availability of single-cell data, comprehensive information on PDE gene distribution within the epididymis and testis has become accessible. Nevertheless, research has primarily focused on cAMP-hydrolyzing PDE isoforms (e.g., PDE4A, PDE4C, PDE7B, PDE8A), which play pivotal roles in testicular germ cells and spermatozoa, while PDE1C—capable of degrading both cAMP and cGMP—has also garnered attention. Nonetheless, growing evidence points to key functions of PDE3 and PDE11 in male fertility. PDE3, especially the PDE3A isoform, has been implicated in sperm function and epididymal contractility, and has potential roles in gametogenesis [43,44,45]; PDE11, in turn, is recognized for its expression in testis, prostate, and developing spermatozoa, influencing spermatogenesis, sperm physiology, and possibly tumor susceptibility [46,47]. In total, at least four PDE-encoding genes have been identified in testicular somatic and germ cells, producing a range of isoforms essential for normal testicular function [48]. Cell-type specificity is also evident: PDE1 and PDE2 localize predominantly to germ cells, whereas PDE3 and PDE4 are found in Sertoli cells. The presence of testis-specific PDE3 mRNAs with unique untranslated regions further highlights the specialized roles that PDEs play in these support cells [48,49,50].

In terms of other PDE isoforms, PDE10A has been detected in human testis and in spermatozoa of certain species, demonstrating variable expression patterns and potential species-specific or developmental stage-dependent functions. Moderate levels of PDE10A immunoreactivity have been observed in canine round and elongated spermatids, whereas its expression is minimal in mature spermatocytes and entirely absent in the epididymides of both rats and humans. Although PDE10A knockout mice suggest that this enzyme may not be crucial for fertilization, its exact role in spermatogenesis remains unclear [32,51]. Additionally, PDE5 expression in rat peritubular myoid cells points to a role for cGMP signaling in contractile functions within the seminiferous tubules [52], and PDE11 has garnered clinical interest due to evidence linking it to testicular function [53].

### 3.1. Phosphodiesterases and cGMP Regulation in the Testis

#### 3.1.1. PDEs in Myoid Cells and the Peritubular Lamina Propria

Through the use of immunohistochemical and electron microscopy techniques, researchers have identified both smooth muscle cells and myofibroblasts as contractile components within the tunica albuginea, along with significant levels of cGMP-binding proteins, such as PKG I [54]. Experiments conducted on isolated tunica albuginea strips have shown that atrial natriuretic peptide (ANP) and the NO donor sodium nitroprusside (SNP) enhance the activity of two cGMP-generating enzymes: GC-A, the receptor for ANP, and soluble guanylate cyclase (sGC) [54]. Interestingly, NO synthase activity was not detected within the contractile cells of the inner tunica albuginea or in Leydig cells. The tunica albuginea exhibits spontaneous contractions primarily in the rete testis region, which can be further stimulated by SNP, cGMP, or ANP. Additionally, SNP has been observed to mitigate noradrenaline-induced contractions across the testicular capsule [54].

The intricate interplay between contraction and relaxation highlights the critical role of guanylate cyclase/cGMP signaling in the functional regulation of the tunica albuginea, ultimately supporting the process of sperm transport. Additionally, ANP has been shown to influence postnatal spermatogenesis [55]. Germ cell-specific cGK-anchoring protein (GKAP42) is expressed in spermatocytes and round spermatids in a stage-specific manner, where it facilitates germ cell development through interactions with PKG Iα [56].

Recent studies have identified GC-A as a key regulator of cell size and proliferation, underscoring the significance of the natriuretic peptide system in developmental processes [57,58]. GC-A also appears to play a role in human sperm function by responding to ANP in a cGMP-dependent manner, which can trigger acrosome reactions. The membrane-associated guanylyl cyclase, GC-B, has been localized in the tunica albuginea, Leydig cells, and peritubular lamina propria. Its ligand, C-type natriuretic peptide (CNP), is uniquely produced by Leydig cells, supporting a paracrine signaling mechanism that targets contractile cells [59]. Research by Middendorff et al. explored the activation of soluble guanylate cyclase (sGC) by carbon monoxide (CO) within human seminiferous tubules. CO, generated by heme oxygenase-1 (HO-1) expressed in Sertoli cells, stimulates sGC activity, resulting in elevated cGMP levels. This CO-induced increase in cGMP within the peritubular lamina propria facilitates the relaxation of myofibroblasts [60,61].

Scipioni et al. reported that PDE5 is present in both Leydig cells and peritubular cells of prepubertal and adult rat testes [33]. Their findings suggest that cGMP-dependent pathways play a crucial role in regulating testosterone production in Leydig cells and contribute to sperm transport through the relaxation of myofibroblasts.

PDE5-mediated cGMP regulation in peritubular myoid cells appears to affect the release of growth factors, such as TGF beta, IGF-I, PModS, and activin-A, as well as extracellular matrix components like proteoglycans, fibronectin, and collagen types I and IV. These factors play a pivotal role in supporting Sertoli cell function and regulating retinol metabolism, which is vital for maintaining the integrity of the germinal epithelium and promoting efficient spermatogenesis. The presence of PDE5 in differentiating and mature peritubular cells in rats indicates its potential importance in myoid cell contractility and in facilitating the overall maturation of the testes [33].

Moreover, chronic administration of PDE5 inhibitors has been associated with structural alterations in testicular tissue, including cyto-architectural degeneration and impaired sperm parameters [62]. These long-term changes may be linked to persistent elevations in cGMP, which can disrupt normal spermatogenesis by inducing apoptosis and affecting epididymal function [62]. Hence, evaluating the impact of PDE5 inhibitors on peritubular cells is crucial for understanding their broader implications for testicular integrity.

#### 3.1.2. PDEs in Leydig Cells

While evidence on the effects of PDE5 inhibitors, such as sildenafil and vardenafil, on sperm characteristics in infertile men remains limited, a study by Dimitriadis et al. explored their impact on Leydig cell secretory function and semen parameters in patients with oligoasthenospermia [63]. The results demonstrated that sildenafil and vardenafil increased Leydig cell activity, indicated by elevated circulating levels of insulin-like factor 3 (Insl3) after treatment. This enhancement in Leydig cell function appears to create a more favorable biochemical environment within the seminiferous tubules, which may have contributed to the improved sperm motility observed in the treated oligoasthenospermic men. Additionally, increased intra-epididymal testosterone concentrations were associated with further improvements in sperm motility and maturation in this patient population [63].

Cyclic adenosine monophosphate (cAMP) plays a central role as the primary mediator of luteinizing hormone (LH)-induced steroidogenesis, primarily through the activation of the cAMP-dependent protein kinase (PKA) pathway [64,65]. Research suggests that non-selective PDE inhibitors can elevate intracellular cAMP levels in Leydig cells, leading to a modest stimulatory effect on testosterone production [66,67]. These findings indicate that several PDE isoforms, beyond PDE5, may influence the intensity, duration, and desensitization of LH-stimulated hormonal responses in Leydig cells [68]. Further studies, such as those by Vasta et al., identified the expression of PDE8A in mouse Leydig cells, where it is implicated in the regulation of steroidogenesis [68]. However, the biological role of PDE8A remains incompletely understood [69,70], particularly as it has also been detected in mouse spermatozoa and human CD4 T lymphocytes, though its specific function in these cells is yet to be clarified [71,72].

In addition, chronic PDE5 inhibitor use may disrupt the hormonal balance by modulating testosterone production over extended periods, potentially impacting Leydig cell function beyond acute stimulatory effects [73]. Prolonged alterations in testosterone homeostasis could also influence estradiol levels, thereby complicating the hormonal environment within the testis [73]. Such disturbances raise concerns regarding possible hypogonadism and reduced spermatogenesis, underscoring the need for further research into long-term PDE5 inhibitor administration [74].

#### 3.1.3. PDEs in Sertoli Cells

Several studies have demonstrated the beneficial effects of PDE5 inhibitors on Sertoli cell secretory function. Vlachopoulou et al. investigated the impact of vardenafil on Sertoli cell secretory function, in both obstructed and non-obstructed azoospermic men. Vardenafil administration in both groups led to increased levels of androgen-binding protein, a key marker of Sertoli cell activity, in testicular cytosol and epididymal fluids, including those from the caput, corpus, and cauda regions [75]. Dimitriadis and colleagues further confirmed the role of PDE5 inhibitors in enhancing Sertoli cell secretory function, potentially by improving peritubular cell function and stimulating the secretory activity of Leydig cells [5,63]. Since testosterone produced by Leydig cells supports Sertoli cell secretory function, improvements in Leydig cell activity may indirectly enhance Sertoli cell performance and, consequently, support spermatogenesis.

However, Scipioni et al. and Dimitriadis et al. reported no evidence for PDE5 expression in Sertoli cells, implying that other PDEs may contribute to cGMP metabolism in these cells [5,33,63]. Research also indicates that cGMP hydrolysis in Sertoli cells is regulated through a calcium–calmodulin-dependent mechanism involving PDE1, which targets both cGMP and cAMP [76]. In addition, PDE3 and PDE4 have been identified in Sertoli cells, emphasizing the intricate nature of PDE regulation within these essential testicular support cells [49,50].

Notably, sustained cGMP elevations due to prolonged PDE5 inhibition can also activate downstream signaling pathways, such as PKG, which have been linked to cell proliferation and apoptosis [77]. Chronic activation of these pathways in Sertoli cells might impair their supportive role in spermatogenesis, further contributing to testicular dysfunction. Therefore, detailed histological assessments are warranted to evaluate whether long-term PDE5 inhibitor use leads to structural disruptions in the seminiferous epithelium.

#### 3.1.4. PDEs in Germ Cells

PDE11 has been shown to be expressed in both human and murine spermatogonia, spermatocytes, and spermatids [78]. Although PDE11 expression is notable and appears to play a role in murine sperm physiology, its precise significance in human sperm function remains uncertain. Studies in PDE11 knockout mice have revealed reduced sperm concentration, impaired motility, and decreased viability, suggesting that PDE11 is crucial for normal fertility [46]. Additionally, PDE1A and PDE10 have been identified in haploid cells of humans and/or mice [52,79]. In situ hybridization and immunofluorescence analyses have revealed prominent expression of PDE1A and PDE1C, though their expression occurs at specific developmental stages of germ cell maturation [79].

More specifically, PDE1A mRNA is detected in round to elongated spermatids, while its protein is localized to the tails of elongated and mature spermatids. Research has confirmed the presence of both PDE1A and PDE1C mRNA and protein in elongating and elongated spermatids (steps 9–16), where transcriptional activity is low, suggesting that PDE1A and possibly PDE1C transcripts are retained for translation during the advanced phases of spermiogenesis. Similarly, PDE3B, PDE4D, and PDE4A have been identified in developing rat spermatocytes, particularly during the pachytene stage [79,80]. Both PDE4A and PDE4D have also been found in haploid cells of rats and mice [80]. Immunofluorescence studies localized the PDE4D protein specifically to the manchette and periacrosomal regions of developing spermatids, a finding confirmed by immunogold electron microscopy. In contrast, PDE4A is predominantly present in the cytoplasm of early-stage haploid male gametes. These observations suggest that PDE4D and PDE4A variants exhibit stage-specific expression and are compartmentalized within distinct subcellular domains during spermatid maturation.

Interestingly, although PDE4D mRNA is readily detected in round spermatids, the corresponding protein is absent at this stage, implying inefficient translation until the later phases of spermiogenesis (steps 11–18). Furthermore, the downregulation of PDE4A variants has been observed in cryptorchid testes, a condition linked to spermatogonia degeneration and increased germ cell apoptosis [81]. Animal model studies have shown that degenerative changes in the testes and alterations in PDE4 expression can be reversed 50 days post-orchidopexy, highlighting the potential reversibility of PDE4-related modifications [81]. Moreover, PDE5 has been identified in ejaculated human spermatozoa, while PDE6 expression has been confirmed in murine spermatozoa at both the RNA and protein levels [71,81].

Importantly, PDE3A has been localized to the postacrosomal segment of the sperm head, suggesting a direct role in sperm function, including motility and capacitation, through cAMP regulation. PDE3B mRNA expression in spermatocytes further implies PDE3 participation in the early stages of gametogenesis [82]. Furthermore, chronic PDE5 inhibitor use may affect other phosphodiesterases, such as PDE6 and PDE11, raising concerns about off-target effects that could disrupt normal germ cell maturation [83]. The potential consequences include compromised sperm morphology, motility, and fertilization capacity, all of which underscore the importance of investigating the safety profile of long-term PDE5 inhibitor therapy [62].

### 3.2. Phosphodiesterase Inhibitors and cGMP Signaling in Male Accessory Genital Glands

#### 3.2.1. The Epididymis

While abnormalities in epididymal contractile function have not been reported in mammalian species, studies suggest that reduced vas deferens contractility can negatively impact male fertility, particularly in mouse models [84,85,86,87]. Despite this, the mechanisms underlying epididymal contraction and relaxation, which are essential for the transport of immotile spermatozoa, remain poorly understood. Evidence to date indicates that cGMP-mediated smooth muscle relaxation plays a role in modulating epididymal duct contractility [88].

Mewe et al. investigated the mechanisms behind spontaneous phasic contractions (SCs) in bovine epididymal tissues using patch-clamping techniques and muscle tension recordings [85]. These contractions, originating from myogenic tissues, were observed in the corpus, caput, and proximal cauda regions of the epididymis. Their maintenance was primarily reliant on extracellular calcium (Ca^2+^) influx via L-type Ca^2+^ channels, with minimal dependence on intracellular Ca^2+^ stores [85].

The role of cGMP in regulating SCs within the bovine epididymal caput and corpus has been explored through immunohistochemical, autoradiographic, and muscle tension analyses [44,89]. Relaxation responses were triggered by sodium nitroprusside (a NO donor), 8-Br-cGMP (a cGMP analogue), and natriuretic peptides ANP and CNP, accompanied by increased immunoreactivity of eNOS, GC-A, and PKG [44]. While cGMP influenced NO, ANP, and CNP activity, the effects of 8-Br-cGMP were modulated by epithelial and luminal components. The administration of L-NAME, a NOS inhibitor, led to increased SC frequency, while PKG inhibition (via Rp-8-Br-cGMPS) and treatments with thapsigargin, iberiotoxin, and indomethacin reduced the impact of 8-Br-cGMP on SCs. These findings indicate that PKG serves as the primary cGMP target, likely acting on SERCA (sarco-endoplasmic reticulum Ca^2+^-ATPase), large-conductance Ca^2+^-activated K(+) channels, and cyclooxygenase-1. PDEs, particularly PDE3, also appear to play a critical role in epididymal peristalsis, as evidenced by studies where milrinone, a selective PDE3 inhibitor, modulated SCs in the epididymal corpus [44,90].

In another study, Dimitriadis et al. found no observable changes in epididymal secretory function following the administration of vardenafil (10 mg daily for 1.5 months) [5]. Similarly, oligozoospermic infertile men treated with 50 mg of sildenafil showed no significant alterations in α-glucosidase levels in semen, an indicator of epididymal function [5]. Research by Müller et al. on Wistar rats revealed that PDE5 expression in the epididymis declines with age but remains unaffected by androgen deprivation [91]. Additionally, Mietens et al. reported that sildenafil significantly decreased the spontaneous contraction rate of the rat epididymal duct [92].

Further research by Alp et al. on Sprague-Dawley rats examined the effects of sildenafil on epididymal semen parameters. Their results showed notable improvements in sperm motility and concentration compared to control groups, suggesting a beneficial effect of sildenafil on epididymal sperm quality [93].

#### 3.2.2. The Vas Deferens

The NO/cGMP phosphodiesterase signaling pathway has been identified as a key regulator of ejaculation. Multiple studies have highlighted the therapeutic potential of sildenafil in treating premature ejaculation [94,95]. For instance, Chen et al. demonstrated that a combination of sildenafil and paroxetine significantly enhanced patient response rates (98%) compared to paroxetine monotherapy (42%), suggesting a synergistic interaction between the two treatments [96].

The relaxing effects of sildenafil on the smooth muscle of the vas deferens may be attributed to elevated cGMP levels. Normally, cGMP export from human erythrocytes relies on ATP and its subsequent breakdown, a process suppressed by PDE inhibitors. By increasing intracellular cGMP concentrations and reducing its hydrolysis and extracellular release, sildenafil facilitates smooth muscle relaxation in the vas deferens [97,98].

Sildenafil is also proposed to influence prejunctional potassium channel activity and reduce adrenergic neurotransmission through NO-independent mechanisms, further promoting vas deferens smooth muscle relaxation [99,100]. Rosevear et al. reported in an experimental study that oral administration of sildenafil (10 mg/kg/day for six months) in Sprague-Dawley rats enhanced the recanalization rate following bilateral vasectomy reconstruction using a biodegradable 5 mm conduit [101].

Similarly, Holoch et al. observed increased recanalization and neovascularization in male rats undergoing vasovasostomy with graft interposition after daily sildenafil treatment (5 mg/kg/day for 16 weeks), though microchannel length remained unchanged [102]. Further evidence by Gur et al. revealed that sildenafil increased the contractile response of the vas deferens to 20 Hz of electrical field stimulation in NO-deficient rats. This effect was negated upon administration of a purinergic (P2X) agonist, suggesting a role for sildenafil in modulating vas deferens contractility [103].

Collectively, these findings indicate that PDE5 inhibitors, such as sildenafil, may play a significant role in regulating vas deferens contractility and, consequently, male reproductive physiology.

#### 3.2.3. The Seminal Vesicles

Dimitriadis et al. examined the impact of sildenafil (50 mg) on seminal vesicle function in men diagnosed with oligozoospermia. Their findings indicated no significant changes in seminal fructose levels, a marker of seminal vesicular secretory activity, across three semen samples collected before and after sildenafil administration. The same investigation evaluated the effect of daily vardenafil (10 mg) in infertile men who had undergone at least one failed in vitro fertilization attempt. Analysis of six semen samples, obtained prior to and during vardenafil treatment, revealed a notable improvement in parameters related to seminal vesicular secretory activity [5].

La Vignera et al. conducted a study to assess ultrasonographic changes in the seminal vesicles of diabetic infertile men following tadalafil treatment (5 mg daily) for three months. Compared to the placebo group, these men demonstrated a significant decrease in the seminal fundus-to-body ratio, an increase in the anteroposterior diameter both pre- and post-ejaculation, enhanced seminal ejection, and substantial improvements in measured sperm parameters [104]. In a previous study, the same researchers reported similar results in 40 infertile patients suffering from male accessory gland infections after receiving tadalafil (5 mg) for three months [105].

Birowo et al. used organ bath experiments to investigate the effects of sildenafil (1 µM for 5 min) on the contractile behavior of smooth muscle cells isolated from human seminal vesicles. The tissues were collected from patients undergoing bladder or prostate surgeries. The results demonstrated that sildenafil suppressed norepinephrine-induced contractions and elevated cGMP concentrations. These findings suggest that tadalafil may also play a role in regulating seminal vesicle contractility [106].

#### 3.2.4. The Prostate

Prostatic secretions consist of citrate, the primary anion essential for maintaining osmotic balance in semen, and a zinc-containing antibacterial complex that plays a key role in protecting chromatin structure within sperm nuclei. Additional prostatic components, such as spermine, are positively associated with sperm motility, while cholesterol is critical for stabilizing sperm membranes against mechanical stress and temperature changes [107]. Sildenafil and vardenafil have been shown to elevate the levels of certain chemicals in semen. This increase in prostatic secretions following sildenafil or vardenafil administration suggests a potential link between these drugs and improved sperm motility [5].

NOS has been detected in both the peripheral and transitional zones of the prostate, particularly near nerve endings within smooth muscle cells. This indicates a role for NO in modulating prostatic smooth muscle tone. Experimental studies have demonstrated that NO donors can promote the relaxation of prostatic smooth muscle tissue [108]. Immunohistochemical evidence has identified PDE4 and PDE5 in the prostate’s transitional zone [109]. Both rolipram, a PDE4 inhibitor, and sildenafil exhibit relaxant effects on smooth muscle strips derived from this zone [109]. These findings emphasize the therapeutic relevance of PDE5 inhibitors and, potentially, PDE4 inhibitors in treating conditions such as benign prostatic hyperplasia and chronic prostatitis [109].

Grimsley et al. proposed that PDE5 inhibitors’ ability to relax prostatic smooth muscle fibers facilitates the clearance of irritative substances from prostatic ducts, reducing inflammation in the prostate [110]. Previous studies have also shown that PDE11 and its isoform PDE11A4 are expressed at high levels in human prostate tissue. The PDE11A gene generates several protein variants through tissue-specific splicing. Müller et al. reported an age-related increase in the expression of GMP-dependent protein kinase I (PRKG1/cGKI) in the prostates of Wistar rats, a finding linked to androgen withdrawal, which contrasts with earlier studies on the epididymis [91]. Yoshinaga et al. demonstrated that administering a single dose of tadalafil improved blood flow in the prostate of a rat model experiencing bladder overdistension and emptying, as well as in a model of aortic clamping and release. These findings suggest that tadalafil may alleviate ischemic–reperfusion damage in the prostate [111].

Das et al. investigated the effects of sildenafil on prostate cancer xenografts in animal models (PC-3 and DU145 cell lines). They reported that sildenafil enhanced the antitumor efficacy of doxorubicin (DOX) by promoting the production of reactive oxygen species (ROS) [112]. Similarly, Gomes et al. demonstrated that chronic sildenafil treatment in mice boosted prostate glandular secretory function and testosterone production without affecting PSA, eNOS, or TGF-β levels [113].

PDE5 inhibitors appear to regulate various biochemical pathways in prostatic tissue. Morelli et al. examined the influence of vardenafil on prostate microcirculation using Doppler ultrasonography in 150 patients with elevated PSA levels (>4 ng/mL) [114]. Their study showed that vardenafil-enhanced power Doppler imaging provided improved visualization of the prostate microvasculature, which may enhance cancer detection rates compared to contrast-enhanced Doppler ultrasound combined with random biopsy methods.

Zhao et al. conducted a clinical investigation on the effects of udenafil and tadalafil on cGMP and cAMP levels in prostate tissue and plasma among 30 patients with erectile dysfunction and lower urinary tract symptoms (LUTSs). These patients later underwent transurethral resection of the prostate (TURP) [115]. Udenafil (200 mg) and tadalafil (20 mg), administered one hour prior to TURP, significantly increased cAMP/cGMP levels, with drug concentrations notably higher in prostate tissue compared to plasma.

Zenzmaier et al. explored tadalafil’s effects on prostate cell lines, revealing its potential utility in managing benign prostatic hyperplasia (BPH) [116]. Their findings showed reduced proliferation of prostatic stromal and epithelial cells, with a greater effect observed on stromal cells. Additionally, tadalafil inhibited the fibroblast-to-myofibroblast transition induced by TGF-β1. Lee et al. evaluated the impact of udenafil on rabbit bladder and prostatic urethra using organ bath techniques, noting dose-dependent relaxation of muscle tissues from both regions, with peak effects at a concentration of 10^−3^ M. These results suggest that udenafil may have therapeutic applications in BPH management [117].

Fibbi et al. examined the role of vardenafil on cultured smooth muscle cells isolated from the prostate, bladder, and urethra of a BPH patient. Their findings indicated that vardenafil enhanced the antiproliferative effects of NO/cGMP pathway activators on bladder and urethral cells, with minimal impact on prostatic cells [118]. Oger et al. investigated the combined effects of tadalafil and alfuzosin on bladder and prostate tissues obtained from cystectomy patients with bladder cancer. In isolated organ bath studies, the combination of tadalafil (10^−6^ M or 10^−5^ M) and alfuzosin (3 × 10^−8^ M, 10^−6^ M, or 10^−5^ M) effectively suppressed adrenergic-induced contractions in prostate smooth muscle [119].

## 4. PDE5 Inhibitors and Semen Quality

### 4.1. Mechanisms of PDE5 Inhibitor Action Regarding Sperm Function

PDE5 inhibitors improve male sexual function by preventing the breakdown of cGMP in the corpus cavernosum. Beyond this localized effect, various PDE isoforms, including PDE5, are expressed in sperm cells, suggesting that PDE5 inhibitors may influence sperm function both directly and indirectly [6]. Several mechanisms have been proposed to explain these effects. Evidence indicates that PDE5 inhibitors can enhance the secretory function of accessory glands, such as the prostate and seminal vesicles, thereby enriching the seminal plasma with factors that support sperm motility, viability, and fertilization potential [120]. By inhibiting PDE5-mediated cGMP hydrolysis, these agents also elevate intracellular cGMP, which can secondarily increase cAMP levels and activate PKA and PKG. These kinases regulate calcium channels, ion fluxes, and signaling pathways essential for initiating and maintaining sperm motility, capacitation, and the acrosome reaction [62,121]. Additionally, PDE5 inhibitors contribute to improving sperm capacitation and acrosome integrity, both of which are critical for successful fertilization [122]. Emerging data suggest that maintaining optimal cyclic nucleotide concentrations through PDE5 inhibition may protect sperm from oxidative stress, enhance mitochondrial function, and increase ATP availability, leading to better motility and extended longevity [123,124]. Non-selective PDE inhibitors like pentoxifylline and caffeine have historically been known to improve sperm motility, and PDE5 inhibitors likely exert similar benefits by modulating related PDE isoforms and downstream targets, thereby optimizing intracellular signaling for normal sperm physiology [125,126]. Furthermore, PDE5 inhibitors support Leydig cell testosterone production and stimulate Sertoli cell secretory functions, creating a favorable biochemical microenvironment for spermatogenesis [40]. These agents may also improve testicular and epididymal contractility, facilitating sperm transport within the male reproductive tract [5].

**Figure 2:**
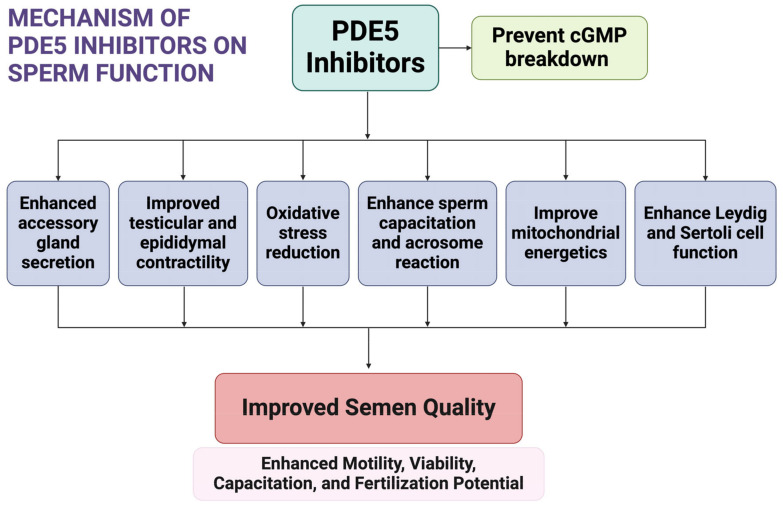
Mechanisms of PDE5 inhibitors regarding sperm function. Created in BioRender (https://BioRender.com/z70m953, accessed on 13 January 2025).

Figure 2 summarizes the mechanisms by which PDE5 inhibitors modulate sperm function, highlighting their effects on accessory gland secretions, intracellular signaling, oxidative stress protection, mitochondrial function, and sperm transport.

### 4.2. In Vivo Evidence

In vivo investigations have explored both non-selective and selective PDE inhibitors to evaluate their effects on semen parameters and overall male reproductive health. Early research focused on non-selective methylxanthine derivatives, such as caffeine and pentoxifylline, which demonstrated improvements in sperm motility dating back to the 1970s [127,128,129,130]. More recent studies have shifted attention to selective PDE5 inhibitors (e.g., sildenafil, vardenafil, tadalafil), producing variable results. While several well-controlled trials reported no significant changes in sperm count, morphology, or motility in healthy or oligozoospermic men, other investigations noted modest increases in sperm motility and total sperm count under certain conditions [5,131,132,133,134,135,136,137,138,139,140,141]. These discrepancies may reflect differences in dosage, treatment duration, patient selection, and the influence of enhanced sexual arousal and improved seminal fluid composition.

Recent genetic studies using Mendelian randomization provide novel insights into the role of PDE5 inhibitors in male reproductive health. Specifically, randomization of genetic variants of PDE5 inhibition was associated with an increase in the number of children fathered by male participants, supporting the potential benefits of PDE5 inhibitors for fertility outcomes. However, no significant associations were found with sexual behavior outcomes or subjective well-being in males, nor were similar effects observed in females. These findings highlight that the reproductive benefits of PDE5 inhibition may be mediated primarily through improved erectile function, which facilitates successful conception. Despite these promising observations, the study emphasizes the need for further clinical investigations to validate these genetic findings and to explore the implications of PDE5 inhibitor use in clinical practice [142].

Moderate doses of PDE5 inhibitors have demonstrated the ability to boost sperm motility and viability; however, higher doses may impair sperm function, potentially due to off-target effects on PDE11 isoforms, which play a critical role in spermatogenesis [143]. Meta-analyses further indicate that PDE5 inhibitors significantly enhance sperm motility, progressive movement, and sperm morphology in infertile men, while having limited effects in individuals with normal semen parameters [144]. These findings underscore the nuanced and dose-dependent nature of PDE5 inhibitors’ impact on male reproductive health. Recent reviews and meta-analyses concluded that oral PDE5 inhibitors enhance sperm motility in men facing infertility [142]. Table 1 summarizes the findings from key meta-analyses, highlighting the effects of PDE5 inhibitors on sperm parameters and their potential implications for male reproductive health.

Clinical observations suggest that PDE5 inhibitors, by improving erectile function, may indirectly benefit couples experiencing infertility related to erectile dysfunction. Their use in male partners of infertile couples has been associated with improved semen collection and reduced stress during fertility treatments [145]. In cases of premature ejaculation, PDE5 inhibitors have been shown to enhance post-ejaculatory penile rigidity, potentially supporting more effective sperm delivery during intercourse [146].

Animal research has yielded similarly mixed findings. Some studies indicate that selective PDE5 inhibitors, like tadalafil, do not adversely affect spermatogenesis or hormone profiles, whereas others report negative effects on testicular architecture and sperm quality when administered at higher doses or for prolonged periods [147,148,149]. Furthermore, investigations in rodent models suggest that PDE5 inhibitors can alter hormonal balance; some data report elevated testosterone levels following sildenafil exposure, while others note increased aromatase activity leading to higher estrogen levels [73]. Additionally, improved vascular supply to the reproductive tract has been observed in diabetic rat models treated with sildenafil, implying potential benefits for sperm function and male fertility [150].

The use of PDE5 inhibitors has been suggested to facilitate outcomes of assisted reproductive technologies (ARTs) by enhancing sperm delivery efficiency and alleviating the psychological stress associated with semen collection, thereby supporting overall treatment success [151].

Overall, the in vivo evidence remains inconclusive. While certain studies highlight the potential for PDE5 inhibitors to improve semen parameters and support fertility, others raise concerns about hormonal alterations and long-term safety. Further research is required to determine optimal dosing, treatment duration, and patient populations, as well as to clarify the complex interactions between PDE5 inhibitors and male reproductive physiology.

### 4.3. In Vitro Evidence

In vitro investigations have largely focused on sildenafil and its direct effects on sperm function. Early studies observed no detrimental impact on sperm viability or motility at clinically relevant concentrations, although very high doses could reduce motility, possibly due to medium acidification [152,153,154]. Later research discovered a sperm-specific PDE5 ortholog in sea urchin spermatozoa, implicating PDE5 in flagellar function and suggesting that its inhibition could enhance sperm motility [153]. Studies have shown that moderate concentrations of sildenafil or tadalafil can improve sperm motility, especially in asthenozoospermic samples, whereas higher doses may have the opposite effect [155,156,157,158,159].

These findings imply that an optimal therapeutic window may exist for PDE5 inhibitors to positively influence sperm motility. Elevated concentrations might induce adverse effects, possibly through off-target inhibition of other PDEs, such as PDE11, which is expressed in testicular tissue and developing sperm cells. Evidence from PDE11 knockout mouse models has indicated that this enzyme may be essential for normal spermatogenesis and fertility, underscoring the potential complexity of PDE interactions in sperm physiology [46].

Current in vitro evidence suggests a dose-dependent effect of PDE5 inhibitors like sildenafil and tadalafil on sperm motility. Moderate doses may enhance motility and potentially support sperm function, whereas higher doses could be detrimental. Further research is warranted to elucidate the precise mechanisms through which PDE5 inhibitors modulate sperm physiology and to determine clinically relevant dosing strategies that optimize benefits while minimizing risks.

### 4.4. PDE5 Inhibition in Sperm Capacitation and Functional Assays

The influence of sildenafil, a PDE5 inhibitor, on the capacitation potential of spermatozoa has been thoroughly examined [155]. Researchers have reported that various concentrations of sildenafil (30 µmol/L, 100 µmol/L, and 200 µmol/L) could initiate the capacitation process in washed spermatozoa. Capacitated sperm were responsive to lysophosphatidylcholine (LPC), either alone or in combination with IBMX—a non-selective PDE inhibitor—by undergoing the acrosome reaction. However, this response was not observed with sildenafil alone, suggesting that PDE inhibitors alone may not trigger or amplify the acrosomal reaction in capacitated spermatozoa [155].

In a separate study, Cuadra et al. examined the effects of varying concentrations of sildenafil (0 nmol/L to 40 nmol/L) on the sperm acrosome reaction [156]. They found that sildenafil significantly influenced the acrosomal response, increasing the proportion of acrosome-reacted spermatozoa by approximately 50% compared to control samples. Since cGMP has been shown to activate cyclic nucleotide-gated channels and facilitate calcium influx into spermatozoa, thereby triggering the acrosome reaction, the inhibitory effect of sildenafil on PDE5, which hydrolyzes cGMP, can augment cGMP-mediated enhancement of the acrosome response [160].

Glenn and colleagues also investigated the impact of sildenafil on the acrosomal reaction. Sperm samples treated with 0.67 µmol/L sildenafil displayed a significant increase in the percentage of acrosome-reacted spermatozoa. In a separate case report, sildenafil was employed to facilitate semen collection for ART procedures [161]. Despite the use of sildenafil, no intracytoplasmic sperm injection attempts resulted in fertilization, potentially due to the older age of the oocytes, caused by delays in semen collection. However, an adverse effect of sildenafil on sperm function cannot be entirely ruled out.

Additional studies have assessed the in vitro effects of PDE5 inhibitors in various sperm functional tests. For instance, Burger et al. investigated the influence of sildenafil on sperm membrane integrity and functional capacity in men with normal fertility and those with infertility. Their findings indicated a significant reduction in membrane integrity in spermatozoa from men with infertility exposed to sildenafil [154]. These results underscore the necessity of caution when considering sildenafil treatment in subfertile couples. In contrast, data from sperm penetration assays suggested that sildenafil exerts minimal influence on those outcomes [154].

## 5. Limitations, Research Gaps, and Future Directions

Although research on PDE5 inhibitors has expanded, the current literature remains limited by methodological heterogeneity and the under-representation of key patient populations. Existing studies vary in their primary endpoints, treatment durations, and dosing regimens, making meaningful comparisons difficult and precluding the formation of standardized clinical guidelines [40]. Many investigations have focused on men without a clear infertility diagnosis, often overlooking those with confirmed subfertility or oligospermia. This omission reduces the clinical relevance of findings and hinders the development of tailored therapies for couples seeking fertility interventions [4]. Moreover, small sample sizes and short follow-up periods undermine the reliability and generalizability of data, rendering it challenging to draw definitive conclusions on long-term effects on spermatogenesis, sperm maturation, or hormonal balance [6]. A further challenge lies in the limited translation of mechanistic data from animal models and in vitro work to human physiology, as interspecies differences may not accurately reflect human reproductive biology [21].

Beyond these broad issues, several more targeted research gaps remain. Key mechanistic questions include how PDE5 inhibitors interact with the hypothalamic–pituitary–gonadal axis, modulate intracellular signaling cascades within Sertoli and Leydig cells, and influence critical processes such as sperm capacitation and the acrosome reaction [4]. Efforts to address these questions must incorporate biomarkers of oxidative stress, mitochondrial health, and genomic stability to clarify whether PDE5 inhibitors exert net positive or negative influences on sperm function and reproductive outcomes [16]. Given the diversity of confounding factors, from age and comorbidities to lifestyle and environmental exposures, standardized protocols and more stringent inclusion criteria are needed to isolate the effects of PDE5 inhibition from other variables [162].

To advance the field, a multipronged approach is essential. Future prospective randomized controlled trials should specifically enroll men with clinically defined fertility issues, employ uniform dosing protocols, and measure standardized endpoints, including sperm parameters and fertilization potential. Longitudinal follow-up is critical to determine whether acute improvements in semen quality translate into sustained fertility benefits and improved pregnancy rates [163]. Comparative studies examining different PDE inhibitor classes and treatment schedules could refine therapeutic precision. Stratification of study populations by demographic and clinical factors (e.g., patient age, baseline semen quality, comorbidities) would help identify subgroups most likely to benefit from PDE5-based interventions [164].

Technological innovations offer promising avenues for addressing these gaps. High-throughput “omics” approaches can delineate molecular networks influenced by PDE5 inhibitors, while advanced imaging techniques can track real-time changes in sperm motility and morphology [165,166]. Computational modeling and artificial intelligence (AI)-driven analyses can integrate complex datasets, ranging from genetic and proteomic profiles to clinical parameters, and predict individual responses to PDE5 therapy. AI-guided biomarker discovery could accelerate the identification of reliable indicators of treatment efficacy, paving the way for personalized, evidence-based treatment plans [167]. By integrating cutting-edge technologies with standardized research designs, the field can move toward gaining a deeper understanding of PDE5 inhibitors’ role in male reproductive health, driving evidence-based clinical applications and enhanced fertility outcomes.

## 6. Conclusions

PDE5 inhibitors, originally developed for the treatment of erectile dysfunction, have emerged as agents with significant potential in modulating male reproductive physiology. Through the regulation of cGMP signaling, PDE5 inhibitors influence multiple aspects of male reproductive health, including sperm motility, capacitation, and Leydig cell function. Evidence suggests that these agents may improve sperm quality and accessory gland function, creating a favorable biochemical environment for spermatogenesis and sperm maturation. However, findings remain mixed, with some studies indicating modest improvements in semen parameters, while others raise concerns about potential adverse effects, particularly with chronic or high-dose use. The mechanisms underpinning these effects likely involve the interplay between intracellular signaling pathways, hormonal balance, and oxidative stress, highlighting the complexity of PDE5 inhibitors’ role in male fertility.

Despite advancements in understanding PDE5 inhibitors, the literature exhibits significant gaps and limitations, including heterogeneity in study designs, small sample sizes, and a lack of standardized outcomes. Much of the current research focuses on healthy men or those with erectile dysfunction, leaving critical questions about their effects in subfertile populations unanswered. Long-term safety data, particularly regarding spermatogenesis, hormonal homeostasis, and epididymal function, remain sparse. Future research must address these gaps through well-designed randomized controlled trials, mechanistic studies, and the application of advanced technologies such as biomarkers and imaging techniques. By overcoming these limitations, a clearer picture of the role of PDE5 inhibitors in male reproductive health will emerge, offering evidence-based guidance for their integration into fertility treatments and broader clinical practice.

## Figures and Tables

**Table 1:** Summary of meta-analyses on PDE5 inhibitors and sperm parameters.

Study Info	Outcome	Infertile Men	Normozoospermic Men
2017 Tan et al. *N* = 1317 [143]	Semen Volume	No significant effect (MD = 0.08; 95% CI: –0.25 to 0.40; *p* = 0.65)	No significant effect (MD = 0.08; 95% CI: –0.47 to 0.64; *p* = 0.76)
	Sperm Concentration	No significant effect (MD = 2.02; 95% CI: –4.43 to 8.47; *p* = 0.54)	No significant effect (MD = 1.36; 95% CI: –5.16 to 7.88; *p* = 0.68)
	% Motile Spermatozoa	Significant increase (MD = 6.89; 95% CI: 3.55–10.23; *p* < 0.001)	No significant effect (MD = 0.67; 95% CI: –2.83 to 4.17; *p* = 0.71)
	% Total Progressive Motility	Significant increase (MD = 6.64; 95% CI: 2.55–10.74; *p* = 0.001)	No significant effect (MD = 2.11; *p* > 0.05) (95% CI not explicitly reported)
	% Rapid Progressive Motility	Significant increase (MD = 3.89; 95% CI: 0.17–7.61; *p* = 0.04)	No significant effect (MD = 0.92; 95% CI: –2.45 to 4.28; *p* = 0.59)
	% Morphologically Normal Spermatozoa	Significant increase (MD = 12.15; 95% CI: 5.16–19.15; *p* = 0.0007)	No significant effect
	Reproductive Hormones	No significant effect on total T, free T, LH, or FSH levels	No significant effect on total T, free T, LH, or FSH levels
2021 Dong et al. *N* = 1121 [144]	Semen Volume	Contradictory findings	Not analyzed separately
	Sperm Number (WHO 1999/2010)	Contradictory findings	Not analyzed separately
	Sperm Concentration (WHO 1999)	Significant improvement (MD = 1.96; 95% CI: 1.70–2.21; *p* < 0.001)	Not analyzed separately
	Sperm Concentration (WHO 2010)	Significant improvement (MD = 3.22; 95% CI: 1.96–4.48; *p* < 0.001)	Not analyzed separately
	% Straight Progressive Motility (Grade A, WHO 1999)	Significant improvement (MD = 3.71; 95% CI: 2.21–5.20; *p* < 0.001)	Not analyzed separately
	Total Sperm Motility (WHO 1999)	Significant improvement (MD = 8.09; 95% CI: 7.83–8.36; *p* < 0.001)	Not analyzed separately
	Progressive Motile Sperm (WHO 2010)	Significant improvement (MD = 5.34; 95% CI: 3.87–6.81; *p* < 0.001)	Not analyzed separately
	% Morphologically Normal Spermatozoa (WHO 1999)	Significant improvement (MD = 0.67; 95% CI: 0.20–1.15; *p* = 0.005)	Not analyzed separately
	% Morphologically Normal Spermatozoa (WHO 2010)	Significant improvement (MD = 1.27; 95% CI: 0.02–2.52; *p* = 0.05)	Not analyzed separately
	Sperm Abnormalities (%) (WHO 1999)	Significant improvement (MD = –0.64; 95% CI: –0.81 to –0.47; *p* < 0.001)	Not analyzed separately

N: sample size; MD: mean difference; CI: confidence interval; *p*: *p*-value; %: percentage; WHO: World Health Organization; LH: luteinizing hormone; FSH: follicle-stimulating hormone.

## Data Availability

Not applicable.
